# Prevalence of Plasmid-Mediated Quinolone Resistance Genes among Extended-Spectrum ***β***-Lactamase-Producing *Klebsiella pneumoniae* Human Isolates in Iran

**DOI:** 10.1155/2015/434391

**Published:** 2015-11-04

**Authors:** Ehsaneh Shams, Farzaneh Firoozeh, Rezvan Moniri, Mohammad Zibaei

**Affiliations:** ^1^Department of Microbiology and Immunology, School of Medicine, Kashan University of Medical Sciences, P.O. Box 8715988141, Kashan, Iran; ^2^Department of Parasitology and Mycology, School of Medicine, Alborz University of Medical Sciences, P.O. Box 3149779453, Karaj, Iran

## Abstract

The purpose of this study was to determine the prevalence and molecular characterization of plasmid-mediated quinolone resistance (PMQR) genes (*qnrA, qnrB, qnrS, aac(6′)-Ib-cr*, and *qepA*) among ESBL-producing *Klebsiella pneumoniae* isolates in Kashan, Iran. A total of 185 *K. pneumoniae* isolates were tested for quinolone resistance and ESBL-producing using the disk diffusion method and double disk synergy (DDST) confirmatory test. ESBL-producing strains were further evaluated for the *bla*_CTX-M_ genes. The PCR method was used to show presence of plasmid-mediated quinolone resistance genes and the purified PCR products were sequenced. Eighty-seven ESBL-producing strains were identified by DDST confirmatory test and majority (70, 80.5%) of which carried *bla*_CTX-M_ genes including CTX-M-1 (60%), CTX-M-2 (42.9%), and CTX-M-9 (34.3%). Seventy-seven ESBL-producing *K. pneumoniae* isolates harbored PMQR genes, which mostly consisted of *aac(6′)-Ib-cr* (70.1%) and *qnrB* (46.0%), followed by *qnrS* (5.7%). Among the 77 PMQR-positive isolates, 27 (35.1%) and 1 (1.3%) carried 2 and 3 different PMQR genes, respectively. However, *qnrA* and *qepA* were not found in any isolate. Our results highlight high ESBL occurrence with CTX-M type and high frequency of plasmid-mediated quinolone resistance genes among ESBL-producing *K. pneumoniae* isolates in Kashan.

## 1. Introduction

*Klebsiella pneumoniae* (*K. pneumoniae*) is one of the most common causes of nosocomial infections in adults and children and the mortality rate because it is high [1, 2]. Prolonged hospitalization, protracted admission in Intensive Care Unit (ICU), long term exposure to antibiotics especially to third-generation cephalosporins, and residing invasive devices are among the risk factors that have been reported for acquisition of infections due to extended-spectrum *β*-lactamases- (ESBL-) producing* K. pneumoniae* in adults [2]. In a study, female sex, corticosteroid therapy, infection with* Klebsiella* species, and recent exposure to third-generation cephalosporins have been reported as the relevant risk factors for acquisition of bloodstream infections with ESBL-producing organisms in children [3]. ESBL-positive* K. pneumoniae* is often resistant to non-*β*-lactam antibiotics (especially fluoroquinolones), so treatment of infections due to these multidrug-resistant strains appears to be very difficult [4, 5]. CTX-M (“CTX” implies activity against cefotaxime, “M” refers to Munich) producing* K. pneumoniae* is becoming increasingly prevalent in many countries of the world [5]. Fluoroquinolone resistance among Gram-negative pathogens developed shortly after these antimicrobials were introduced [6, 7].

Although the preliminary mechanism of fluoroquinolone resistance in members of the family Enterobacteriaceae is traditionally mediated by the mutation of chromosomal genes encoding DNA gyrase, topoisomerase IV, regulatory efflux pumps, and/or porins, recent reports indicate that quinolone resistance may also be related to plasmid-mediated quinolone resistance genes (*qnrA*,* qnrB*,* qnrS*,* aac(6*′*)-Ib-cr,* and* qepA*) [8, 9]. The quinolones inhibit DNA gyrase as their target site of action and cause bacterial cell death. Quinolone resistance genes including* qnrA*,* qnrB*, and* qnrS* code for proteins belonging to the pentapeptide repeat family interacting with DNA gyrase and topoisomerase IV enzymes to prevent quinolone inhibition [6]. The second PMQR mechanism captures a variant of aminoglycoside acetyltransferase (AAC(6′)-Ib-cr), which can diminish a fluoroquinolone activity by adding an acetyl group to this antimicrobial agent [10]. The last mechanism of PMQR is the quinolone efflux pump (QepA), a proton-dependent transporter, which causes hydrophilic quinolone resistance, especially to norfloxacin, ciprofloxacin, and enrofloxacin [9]. Plasmid-mediated quinolone resistance (PMQR) determinants have been recognized worldwide with a thoroughly high prevalence among extended-spectrum *β*-lactamases- (ESBLs-) producing* K. pneumoniae* [1, 6, 7, 11–13]. Little is known about the frequency of PMQR genes in* K. pneumoniae* isolates recovered from clinical specimens from humans in Iran. Therefore, the aim of this study was to determine the prevalence of PMQR genes in ESBLs-positive* K. pneumoniae* isolated from clinical specimens of patients referred to Shahid Beheshti hospital in Kashan, Iran.

## 2. Material and Methods

### 2.1. Sample Collection

A cross-sectional study was conducted between April 1, 2013, and April 17, 2014. One hundred eighty-five nonrepetitive clinical isolates (urine, blood, wound, sputum, etc.) suspected to be* K. pneumoniae* and isolated from patients referred to Shahid Beheshti Hospital in Kashan were tested in the study. Approval of the study protocol was received from the Ethical Review Board of Kashan University of Medical Sciences. Written informed consent was obtained from all study participants or their parents/guardians. Of the patients that participated in the study, 114 (61.6%) were females and 71 (38.4%) were males.

### 2.2. Bacterial Isolates

One clinical specimen was obtained for culture and microbial characterization from each patient and was used for bacterial culture. Each specimen was immediately inserted into Tryptic Soy Broth (TSB) medium (Merck, catalogue number: 105459) and sent to laboratory and, after incubation at 37°C for 6–12 hours, cultured on MacConkey agar (Merck, catalogue number: 105465) for the isolation of bacteria.

The cultured plates were incubated at 37°C and were examined after overnight incubation. Characteristic colonies of* K. pneumoniae* were confirmed by gram staining, oxidase, and biochemical tests including Indole, Methyl Red, Voges Proskauer, and Citrate (IMVIC) tests [14]. The confirmed* K. pneumoniae* isolates were used for study of resistance pattern and DNA extraction.

### 2.3. Antimicrobial Susceptibility Testing and Determination of ESBL Production

Antimicrobial susceptibility and detection of quinolone resistance were determined by disk diffusion method using Mueller Hinton agar according to the Clinical and Laboratory Standards Institute (CLSI) recommendations [15]. The following disks were used: imipenem (*S*: ≥16 mm, *I*: 14-15 mm, and *R*: ≤13 mm), aztreonam (*S*: ≥21 mm, *I*: 18–20 mm, and *R*: ≤17 mm), ampicillin (*S*: ≥17 mm, *I*: 14–16 mm, and *R*: ≤13 mm), ciprofloxacin (*S*: ≥31 mm, *I*: 21–30 mm, and *R*: ≤20 mm), cephalothin (*S*: ≥18 mm, *I*: 15–17 mm, and *R*: ≤14 mm), cefotaxime (*S*: ≥26 mm, *I*: 23–25 mm, and *R*: ≤22 mm), cefoxitin (*S*: ≥18 mm, *I*: 15–17 mm, and *R*: ≤14 mm), ceftriaxone (*S*: ≥23 mm, *I*: 20–22 mm, and *R*: ≤19 mm), gentamicin (*S*: ≥15 mm, *I*: 13-14 mm, and *R*: ≤12 mm), nalidixic acid (*S*: ≥19 mm, *I*: 14–18 mm, and *R*: ≤13 mm), ceftazidime (*S*: ≥21 mm, *I*: 18–20 mm, and *R*: ≤17 mm), and amoxicillin*-*clavulanic acid (*S*: ≥18 mm, *I*: 14–17 mm, and *R*: ≤13 mm). The reference strain* E. coli* ATCC 25922 was used as a control. Results were interpreted as susceptible, intermediate, or resistant according to the criteria recommended by the CLSI and the manufacturer protocols (Mast, UK). Double disk synergy test was accomplished in strains which were resistant to ceftriaxone, ceftazidime, cefotaxime, and aztreonam by disk diffusion method to confirm production of extended-spectrum *β*-lactamases (ESBLs) using ceftazidime discs with and without clavulanic acid, which were placed on the center of a plate containing Mueller Hinton agar medium at a distance of 25 mm from each other [16].

### 2.4. DNA Extraction

Crude DNA was extracted from each* K. pneumoniae* isolate extracted from the bacterial suspension by boiling method. The template DNA stored at −20°C until polymerase chain reaction (PCR) amplification was performed.

### 2.5. Detection of ESBL CTX-M Type by PCR

*K. pneumoniae* isolates that were ESBL-positive by DDST confirmatory test were subjected to amplification by PCR method using *bla*_CTX-M_ specific primers (Table 1). Final reaction mixtures volume of 25 *μ*L was prepared with 10 pmol of each primer, 200 mM of dNTP, 1 unit of Taq polymerase, 2.5 *μ*L of 10x reaction buffer, 1.5 mM MgCl_2_ in final concentration, and 100 ng DNA template. Amplification reactions were carried out in a thermocycler (Eppendorf master cycler, MA) under the following conditions: 94°C for 5 min, followed by 30 cycles of 94°C for 25 sec, 52°C for 40 sec, 72°C for 50 sec, and 72°C for 6 min for the final elongation step [17]. The amplified products were electrophoresed on 2% agarose gels. The gels were stained in ethidium bromide (0.5 mg/mL) visualized in gel document system (Biorad, UK).

### 2.6. Characterization of PMQR-Harboring Strains

PMQR genes, including* qnrA*,* qnrB*,* qnrS*,* aac(6*′*)-Ib-cr*, and* qepA*, were identified by PCR and Sanger sequencing. Pairs of primers used for PCR and sequencing are shown in Table 1. Amplification for* qnrA*,* qnrB*, and* qnrS* was carried out under the following conditions: initial denaturation at 94°C for 5 min, followed by 32 cycles of amplification at 94°C for 45 sec, 53°C for 45 sec, and 72°C for 60 sec with final extension at 72°C for 5 min [10]. PCR conditions for* aac(6*′*)-Ib-cr* were as follows: initial denaturation at 95°C for 5 min, followed by 32 cycles of amplification at 95°C for 20 sec, 59°C for 40 sec, and 70°C for 30 sec, with final extension at 72°C for 5 min [18].

The amplification conditions used for detection of* qepA* were as follows: initial denaturation at 94°C for 5 min, followed by 30 cycles of amplification at 94°C for 1 min, annealing at 59°C for 30 sec, and extension at 72°C for 1 min, with final extension at 72°C for 5 min [19]. The PCR was performed on total volume of 50 *μ*L containing 100 ng genomic DNA from* K. pneumoniae* culture, 200 mM each of dNTP, 1x PCR buffer (20 mM Tris-HCl, pH 8.4), 50 mM KCl, 1.5 mM MgCl_2_, 0.5 mM of each primer, and 1.5 U of Taq polymerase. Amplified samples (10 *μ*L) were electrophoresed on 1.0% agarose gel in TBE buffer. The gel was stained with ethidium bromide 0.5 mg/mL. The amplified bands were visualized under ultraviolet light and photographed. Reaction mixtures without a DNA template served as negative controls. All positive amplicons were sequenced to confirm the PMQR genes.

### 2.7. DNA Sequencing and Sequence Analysis

PCR products that contain the positive desired genes (*qnrB*,* qnrS*, and* aac(6*′*)-Ib-cr*) were sequenced using the ABI Capillary System (Macrogen Research, Seoul, Republic of Korea). The sequences were analyzed using Chromas Pro version 1.7.5 Technelysium (http://technelysium.com.au/) and carried out online using the BLAST program of the National Center for Biotechnology Information server (http://www.ncbi.nlm.nih.gov/).

### 2.8. Statistical Analysis

All statistical analyses were carried out using SPSS for windows version 15.0. Differences by the *χ* ^2^ test were considered significant, if *P* < 0.05. Prevalence data is presented with 95% confidence intervals (CI).

## 3. Results

Of the 185* K. pneumoniae* species included in this study, 127 (68.6%) were nosocomial and were isolated from patients who had been hospitalized for two days or more in the Shahid Beheshti Hospital of Kashan and had no signs of infection with* K. pneumoniae* at the beginning of hospitalization.* K. pneumoniae* species were isolated from urine, 119 (64.3%), wound, 9 (4.8%), blood, 6 (3.2%), respiratory tract samples, 46 (24.9%) (including 5 of sputum, 3 of bronchoalveolar lavage, 37 of respiratory chip, and 1 of nasal discharge), cerebrospinal fluid, 2 (1.1%), and catheter, 3 (1.6%).

**Figure 1:**
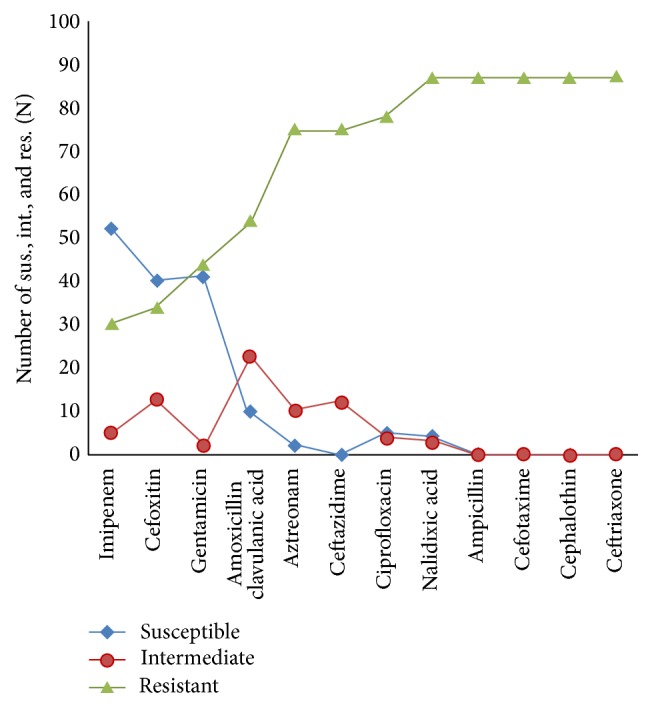
Antimicrobial resistance of ESBL-producing* Klebsiella pneumoniae* isolates was measured by disk diffusion method.

The mean age of the patients was between 48 and 52 years (50.36 ± 3.80). The prevalence rate of ESBL-producing* K. pneumoniae* infection was 47.0% (87/185). Of these ESBL-positive* K. pneumoniae* isolates, 82 (94.3%) were nosocomial. The majority of ESBL-positive patients were female, 48 (55.2%), versus 39 male (44.8%) and the most ESBL-producing strains (% 64.4) were recovered from elderly patients (<50 years of age) whereas the rates of ESBL-producing* K. pneumoniae* isolated from younger patients (≤50 years) were lower (35.6%). Among 87 ESBL-positive* K. pneumoniae* isolates, 78 (89.7%) were fluoroquinolone resistant, of which 100% demonstrated multidrug-resistant (MDR) pattern and were resistant to at least one agent in three or more antimicrobial categories. Among ESBL-positive* K. pneumoniae* isolates, the most prevalence of resistance was seen against ceftriaxone, cephalothin, cefotaxime, and ampicillin (Figure 1). Of isolates that were deemed ESBL-producing bacteria by DDST test, 80.5% (70/87) contained *bla*_CTX-M_ genes. PCR assays and sequencing of *bla*_CTX-M_ genes revealed that 60% (*n* = 42), 42.9% (*n* = 30%), and 34.3% (*n* = 24) of these isolates were identified as CTX-M-1, CTX-M-2, and CTX-M-9, respectively; also none of the isolates were positive for CTX-M-8. PMQR determinants were using PCR in 77 (88.5%) ESBL-positive isolates, which mostly consisted of* aac(6*′*)-Ib-cr* and* qnrB*, followed by* qnrS* genes. Among the PMQR-positive isolates, 30 (39%) and 1 (1.3%) of which coharbored 2 and 3 different PMQR genes, respectively. However,* qnrA* and* qepA* were not found in any isolate (Table 2). The nucleotide sequences of the PCR products of PMQR genes were all identical to those* qnrB1*,* qnrS8*, and* aac(6*′*)-Ib-cr* in the GenBank nucleotide database (http://www.ncbi.nlm.nih.gov/blast/) and accession numbers obtained for them in current study are KP340790, KP340791, and KP340792, respectively.

The statistical analysis confirmed that ICU admission, more than fifty years of age and urinary infection, and infection with fluoroquinolones-resistant and multidrug-resistant strains (*P* < 0.001) were clinical factors that significantly associated with the presence of* K. pneumoniae* isolates that would yield positive results for ESBL molecular testing (Table 3).

## 4. Discussion

Plasmid-mediated quinolone resistance mechanisms play an important role in the expansion of quinolone and fluoroquinolone resistance among ESBL-producing* K. pneumoniae* [20]. Infections caused by such resistant isolates can be difficult to treat [21]. Over the past 10 years, PMQR determinants have appeared as an important issue [22].

In this study, the resistance rates to nalidixic acid and ciprofloxacin were very high. Since the development of nalidixic acid in 1962, the drug has been used for the treatment of infections caused by* K. pneumoniae* [23]. The resistance rate for nalidixic acid, therefore, is expected to be higher than that for fluoroquinolones. Ciprofloxacin is frequently used for treatment of* K. pneumoniae* infections in Iran [24, 25]. Other studies in most parts of the world have indicated that fluoroquinolone resistance in ESBL-producing* K. pneumoniae* isolates is increasing [26]. Although the exact causes of the high ciprofloxacin resistance rate found in the present study compared to previous reports [26–29] are not known, the differences in the culture methodology between studies may explain the differences in the prevalence.

Our results showed the prevalence rates of ESBL-producing* K. pneumoniae* isolates were comparable to those recently conducted in Tehran, Iran [25]. A higher prevalence was noted in Mangalore (48.53%); however, lower prevalence was noted in China (33.3%), Saudi Arabia (12.2%), the United States (8%), and Canada (5%) [22, 26, 30–32].

In terms of association between patient characteristics and acquisition of ESBL-producing* Klebsiella* infection, we found that ESBL production was significantly higher among elderly patients. Similar results have been recorded in studies from Ghana and Saudi Arabia [33, 34]. The increased ESBL prevalence in elderly patients could be due to higher rates of prior antimicrobial therapy in this group of ages that led to greater antibiotic pressure among them. To date,* qnr* genes have been widely discovered in southern and eastern Asia, North and South America, and Europe [10]. Our study demonstrated the high prevalence (51.7%) of* qnr* genes among 87 ESBL-producing isolates of* K. pneumoniae* that were comparable with those noted in Morocco (50.0%) and China (65.5%) [20, 35]. However, low frequencies were found in Tunisia (15.0%), Mexico (13.7%), and USA (11.1%) [6, 13, 32]. In current study, the highest prevalence of* qnr* genes was related to* qnrB*. In Brazil,* qnrB* has been reported as the most widespread* qnr* determinant [36, 37]. No* K. pneumoniae* isolate harbored* qnrA* gene, although the prevalence of this gene was high among* E. coli* isolates in our previous study [24]. The low prevalence of* qnrS* and the absence of* qnrA* observed in our study were in agreement with studies from France and Tunisia [6, 38]. AAC(6′)-Ib-cr, a novel PMQR protein, was first reported in 2003 but is now recognized to be widely distributed. Overall,* aac(6*′*)-Ib-cr* genes were the most frequent PMQR determinant in our study. The high prevalence of* aac(6*′*)-Ib-cr* among* K. pneumoniae* isolates in our region may be related to clonal spread of a single clone, although further studies using molecular typing methods such as pulsed field gel electrophoresis (PFGE) are needed to confirm this statement. The absence of* qepA* observed in this study was comparable to the most recent studies conducted in Thailand, Korea, and Mexico [8, 9, 13]. The* qepA* gene has been documented only from Japan, France, and China and mostly detected among* E. coli* strains [39]. Our finding showed some PMQR-positive* K. pneumoniae* isolates coharbored different PMQR genes. This finding could indicate that these PMQR genes were located on the same plasmid, although plasmid analysis is required to confirm this. The majority (60%) of CTX-M-type ESBL producer isolates were CTX-M-1. In a study in Russian hospitals, in agreement with our findings, 92.9% of CTX-M *β*-lactamases were found to be CTX-M-1 type [17]. Association of* qnr* genes with extended-spectrum* beta*-lactamases (ESBLs) has been described [40]. Our* qnr*-positive ESBL producer* K. pneumoniae* isolate showed high positive rates of* beta*-lactamases such as CTX-M-1 and CTX-M-2. This was primarily attributable to the* qnrB1* subtype. We suspect that* qnr* genes possibly contribute to the widespread distribution of CTX-M-1 and CTX-M-2 in our region where fluoroquinolones are widely used. Our results also revealed that some *bla*_CTX-M_ negative isolates were ESBL-producing showing that ESBL genes of other families may be involved. Another interesting finding was that fluoroquinolone-resistant ESBL-producing* K. pneumoniae* strains which carried* qnrB*,* qnrS*, and* aac(6*′*)-Ib-cr* genes were specifically disseminated in ICU ward.

This result is alarming because hospitalized ICU patients are vulnerable and tend to have more risk factors than the other patients; also selective pressure exerted by extensive antibiotic use in ICU ward favors selection and transmission of such multidrug-resistant strains.

## 5. Conclusion

The results showed high ESBL occurrence with CTX-M type and high frequency of plasmid-mediated quinolone resistance genes among ESBL-producing* K. pneumoniae* isolates in our region. The* aac(6*′*)-Ib-cr* variant and* qnrB* gene were widely distributed among* K. pneumoniae* isolates. The screening of ESBL-producing* K. pneumoniae* for PMQR carriage could be helpful in both treatment and prevention of spread of resistant strains.

## Figures and Tables

**Table 1:** Primers used for polymerase chain reaction and sequencing.

Primer	Target gene	Sequence (5′ → 3′)	Amplification product (bp)	Reference
CTX-MA CTX-MB	*bla*_CTX-M_ *bla*_CTX-M_	CGCTTTGCGATGTGCAG ACCGCGATATCGTTGGT	590	[17]
M-1F M-1R	*bla*_CTX-M-1_ *bla*_CTX-M-1_	AAAAATCACTGCGCCAGTTC AGCTTATTCATCGCCACGTT	415	[17]
M-2 M-2R	*bla*_CTX-M-2_ *bla*_CTX-M-2_	CGACGCTACCCCTGCTAT CCAGCGTCAGATTTTTCAGG	552	[17]
M-8F M-8R	*bla*_CTX-M-8_ *bla*_CTX-M-8_	ACGCTCAACACCGCGATC CGTGGGTTCTCGGGGATAA	490	[17]
M-9F M-9R	*bla*_CTX-M-9_ *bla*_CTX-M-9_	CAAAGAGAGTGCAACGGATG ATTGGAAAGCGTTCATCACC	205	[17]
qnrA F qnrA R	*qnrA* *qnrA*	ATTTCTCACGCCAGGATTTG GATCGGCAAAGGTTAGGTCA	516	[13]
qnrB F qnrB R	*qnrB* *qnrB*	GATCGTGAAAGCCAGAAAGG ACGATGCCTG¬GTAGTTGTCC	469	[13]
qnrS F qnrS R	*qnrS* *qnrS*	ACGACATTCGTCAACTGCAA TAAATTGGCACCCTGTAGGC	417	[13]
aac(6′)-Ib-cr F aac(6′)-Ib-cr R	*aac(*6′*)-Ib-cr* *aac(*6′*)-Ib-cr*	TGACCAACAGCAACGATTCC TTAGGCATCACTGCGTGTTC	554	[14]
qepA F qepA R	*qepA* *qepA*	GGACATCTACGGCTTCTTCG AGCTGCAGGTACTGCGTCAT	720	[15]

**Table 2:** Prevalence of the plasmid-mediated quinolone resistance genes among extended-spectrum *β*-lactamase-producing *Klebsiella pneumoniae* isolates.

PMQR genes	Positive isolates *n* (%)	Negative isolates *n* (%)	Total *n* (%)
*qnrA*	0 (0%)	87 (100%)	87 (100%)
*qnrB*	40 (46.0%)	47 (54.0%)	87 (100%)
*qnrS*	5 (5.7%)	82 (94.3%)	87 (100%)
*aac(*6′*)-Ib-cr*	61 (70.1%)	26 (29.9%)	87 (100%)
*qepA*	0 (0%)	87 (100%)	87 (100%)

**Table 3:** Association between patient characteristics and acquisition of extended-spectrum *β*-lactamase-producing *Klebsiella pneumoniae* infection.

Patient characteristic	ESBL-negative *n* = 98 (%)	ESBL-positive *n* = 87 (%)	*P* value
Age			
>50 years	36 (36.7)	56 (64.4)	≤0.001
≤50 years	62 (63.3)	31 (35.6)	
Sex			
Male	27 (27.6)	39 (44.8)	0.05
Female	71 (72.4)	48 (55.2)	
Ward			
ICU	15 (33.3)	23 (28)	≤0.001
Internal medicine	7 (15.6)	10 (12.2)	
Infectious	5 (11.1)	14 (17.1)	
Surgery	6 (13.3)	12 (14.6)	
Gynecology	4 (8.9)	7 (8.5)	
Pediatric	5 (11.1)	7 (8.5)	
Emergency	1 (2.2)	6 (7.3)	
CCU	2 (4.4)	3 (3.7)	
Sample type			0.001
Urine	74 (75.5)	45 (51.7)	
CSF	1 (1)	1 (1.1)	
Catheters	1 (1)	2 (2.3)	
Blood	5 (5.1)	1 (1.1)	
Wound	3 (3.1)	6 (6.9)	
Respiratory	14 (14.3)	32 (36.8)	
Drug resistance			
Fluoroquinolones resistance	5 (5.1)	78 (89.7)	≤0.001
Multidrug resistance	62 (63.3)	87 (100.0)	
